# Effect of Ag, Sn, and SiCN Surface Coating Layers on the Reliability of Nanotwinned Cu Redistribution Lines Under Temperature Cycling Tests

**DOI:** 10.3390/ma17225458

**Published:** 2024-11-08

**Authors:** Yu-Wen Hung, Mai-Phuong La, Yi-Quan Lin, Chih Chen

**Affiliations:** Department of Materials Science and Engineering, National Yang Ming Chiao Tung University, Hsinchu 300093, Taiwan; patty8862000@gmail.com (Y.-W.H.); phuongla219@gmail.com (M.-P.L.); rex0199522@gmail.com (Y.-Q.L.)

**Keywords:** nanotwinned copper, redistribution lines, passivation layer, reliability, temperature cycling test

## Abstract

Nanotwinned Cu (NT-Cu) is a promising candidate for Cu redistribution lines (RDLs). However, oxidation in NT-Cu lines is of concern because it increases electrical resistance and endangers the reliabilities of semiconductor devices such as temperature cycling tests (TCTs). In order to enhance the reliabilities, the passivation of NT-Cu lines is needed. In this study, immersion Ag/Sn and plasma-enhanced chemical vapor deposition (PECVD) SiCN were used to passivate the surfaces of NT-Cu RDLs at low operating temperatures (60 °C for immersion and 150 °C for PECVD). We found that Ag- and SiCN-capped NT-Cu lines showed negligible changes in microstructures and resistance after TCTs. As for Sn-coated NT-Cu lines, the resistance remained stable after 250 cycles of TCTs, with low oxygen signals detected. These three coating layers can block oxygen and moisture, effectively preventing oxidation and maintaining the resistance of NT-Cu RDLs during the TCT. The findings demonstrate the effectiveness of Ag, Sn, and SiCN coatings in enhancing reliability, providing options for passivation layers of NT-Cu RDLs.

## 1. Introduction

The global race in the development of artificial intelligence has required a huge demand for high-performance computing [1,2]. In response, the semiconductor industry has been pursuing miniaturization, cramming more transistors into smaller chips to enhance chip processing power. Currently, one effective method is to adopt advanced packaging technology for maintaining scaling trends in power, performance, area, and cost (PPAC) [3,4]. In three-dimensional integrated circuit (3D IC) or two-dimensional integrated circuit (2.5D IC) technology, high-density Cu redistribution lines (RDLs) with small line widths are essential for interconnecting different chip components and securing seamless input/output (I/O) data flow [5,6] due to their cost-effectiveness and flexibility [7,8]. For example, replacing the through-Si-via (TSV) interposer or entire polymer substrates with fine-pitch RDLs can reduce manufacturing costs while enhancing chiplet performance [9]. However, downsizing RDL dimensions may deteriorate the reliability of the Cu traces [10]. As the cross-sectional dimensions of the Cu traces decrease, the resulting increase in current density aggravates mass transport [11,12]. This electromigration (EM) phenomenon causes the formation of voids and thus increases electrical resistance. Additionally, during the product lifecycle, the stress from mismatched coefficients of thermal expansion (CTE) between materials may cause mechanical failures in the Cu traces [13,14].

Nanotwinned Cu (NT-Cu) is a promising material in microelectronic packaging industries due to its superior mechanical properties, conductivity, and EM resistance [15,16,17,18,19,20,21,22,23]. A previous study showed that NT-Cu lines deposited on Si substrates could endure thermal stress without cracking during the temperature cycling test (TCT) [13]. However, the resistance change was still observed in the NT-Cu lines, possibly due to oxidation. In the EM studies of NT-Cu lines, it was noted that oxidation of the NT-Cu lines significantly influenced EM lifetime owing to the high resistivity of Cu oxide [24,25]. To mitigate the Cu oxidation, a surface coating layer to passivate Cu lines is employed [11,26,27,28]. Polymers are commonly selected as surface coating layers to cover Cu RDLs. Nevertheless, the curing process of polymers above 200 °C is required. Furthermore, the polymer capping layer exhibits limited resistance to moisture absorption and oxygen permeability, thus facing significant reliability challenges [29,30,31,32,33]. For instance, solvent evaporation during polyimide (PI) curing induces the formation of voids or gaps. These channels act as pathways for oxygen and moisture diffusion, diminishing the antioxidant properties of the PI layer and shortening the EM lifetime of NT-Cu lines [24,25]. Therefore, to improve the reliability of NT-Cu RDLs, it is crucial to explore alternative passivation layer materials that can retard the oxidation of the NT-Cu lines.

Herein, we employed various passivation layers, including immersion Ag/Sn and plasma-enhanced chemical vapor deposition (PECVD) SiCN, to cover the surfaces of the NT-Cu lines with low operating temperatures (60 °C for immersion, 150 °C for PECVD), which helps to reduce the thermal budget. Additionally, this study aims to systematically analyze the resistance variations, void generation, and oxidation in NT-Cu lines coated with these protective layers after the TCT.

## 2. Materials and Methods

**Figure 1:**
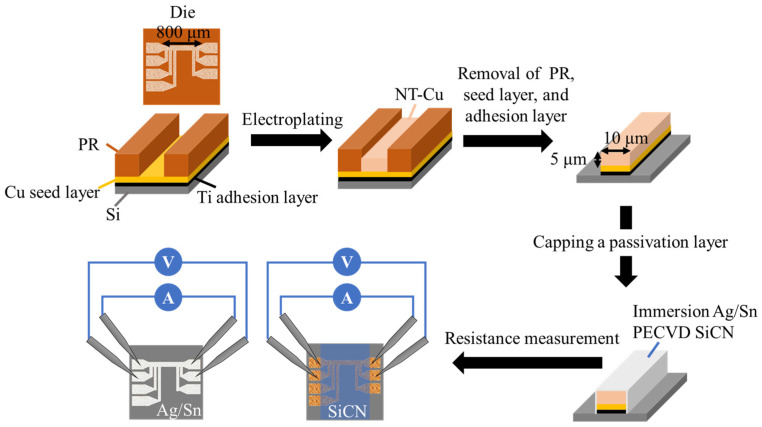
Schematic diagram illustrating the preparation flow and electrical resistance measurement of the NT-Cu RDLs.

The fabrication procedure of NT-Cu RDL samples is illustrated in Figure 1. The RDL die (1.25 cm × 1 cm) contained 100 nm Ti adhesion and 200 nm Cu seed layers, defining a Cu line (800 µm in length) and probing pads. The electroplating solution was prepared with 0.8 M of CuSO_4_, 40 ppm of HCl, 100 g/L of H_2_SO_4_, and additives of model DP101 from Chemleader Corporation (Hsinchu County, Taiwan) for producing nanotwins. The NT-Cu lines were electrodeposited with a thickness of 5 μm and a width of 10 μm using periodic reverse current. The forward and reverse current densities were 4 A/dm^2^ and −1 A/dm^2^, respectively, with on-time and reverse-time durations of 40 ms and 4 ms per cycle. Next, the photoresist (PR), Cu seed layer, and Ti adhesion layer were removed by acetone and commercial etching solution from Chemleader Corporation. The NT-Cu RDL samples were then coated with different types of passivation layers. For Ag or Sn coating, NT-Cu RDL samples were immersed in a commercial electroless plating solution provided by MacDermid Alpha (MacDermid Alpha, Taoyuan City, Taiwan). The commercial electroless plating solutions were used as received, and the temperature of the electroless plating solutions was controlled at 60 °C. For SiCN capping, a SiCN capping layer was fabricated through in-house plasma enhanced chemical vapor deposition (PECVD) at an operating temperature of 150 °C.

**Figure 2:**
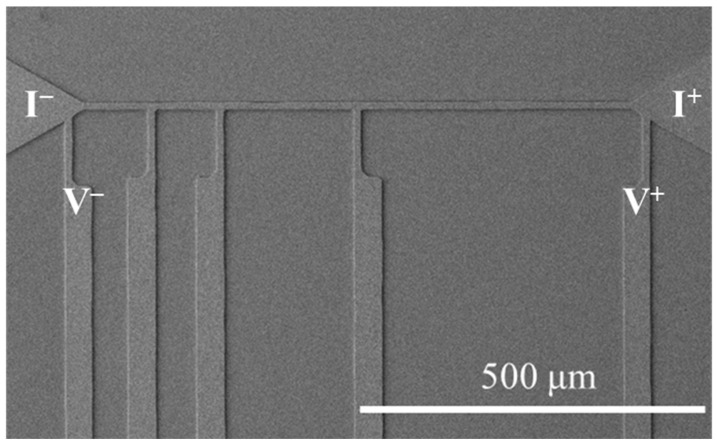
Plan-view SEM image of the NT-Cu line describing the current direction and voltage measurement positions.

The RDL samples were then subjected to the TCT ranging from −55 °C to 125 °C with a dwell time of 5 min and a ramp rate of 18 °C/min. The resistances of RDL samples before and after the TCT were measured using a four-point probe system, as depicted in Figure 1. Figure 2 also illustrates the current direction and the positions for voltage measurement. For the resistance measurements, current was applied between the two upper probing pads, while voltage was measured between the two lower probing pads. Resistance values were then calculated according to Ohm’s law. For each condition, the resistance values of three RDL samples were measured to calculate the average values and analyze the standard deviation. The microstructure was characterized using a focused ion beam (FIB, Helios G3CX, FEI, Hillsboro, OR, USA). Elemental analysis was conducted with energy-dispersive X-ray spectrometry (EDX) within a scanning electron microscope (SEM, JSM-7800F, JEOL Ltd., Tokyo, Japan).

## 3. Results and Discussion

**Figure 3:**
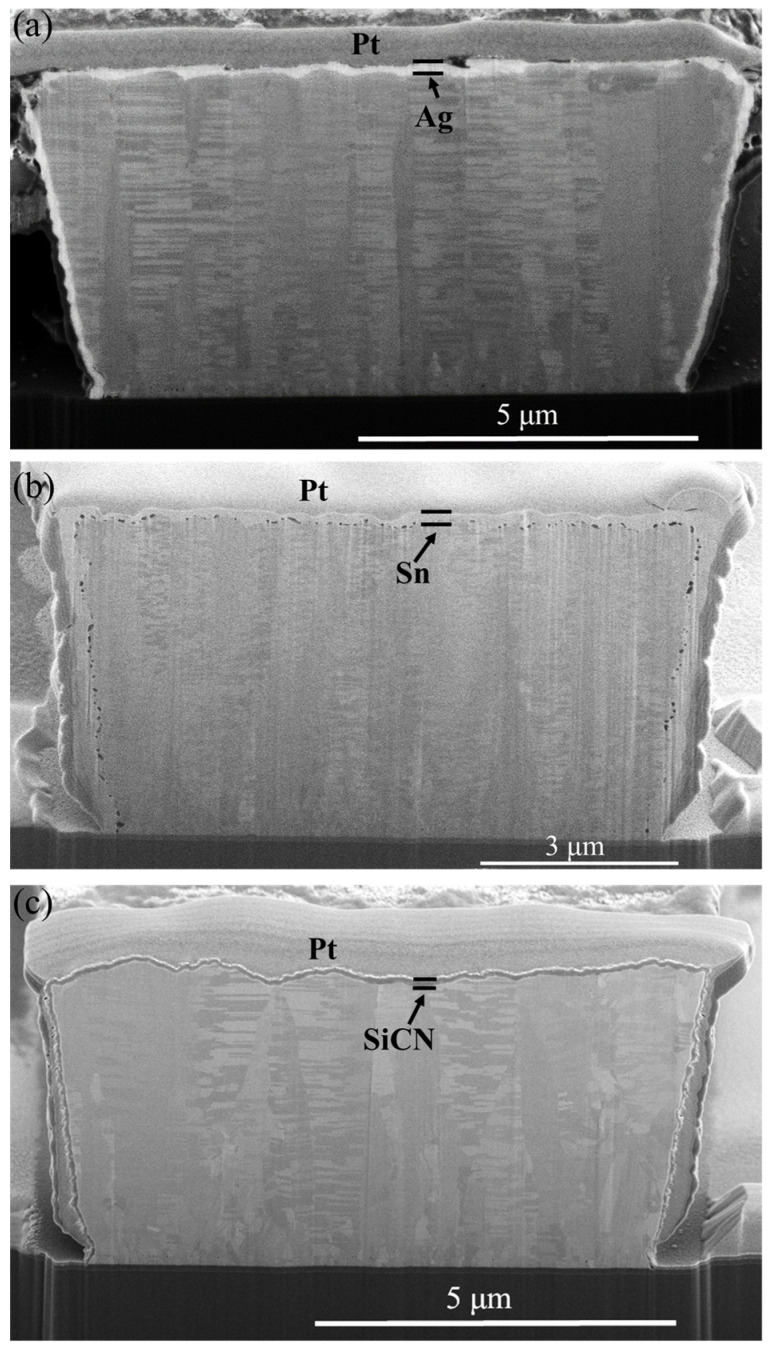
Cross-sections of the as-prepared RDLs coated with (**a**) Ag, (**b**) Sn, and (**c**) SiCN.

**Figure 4:**
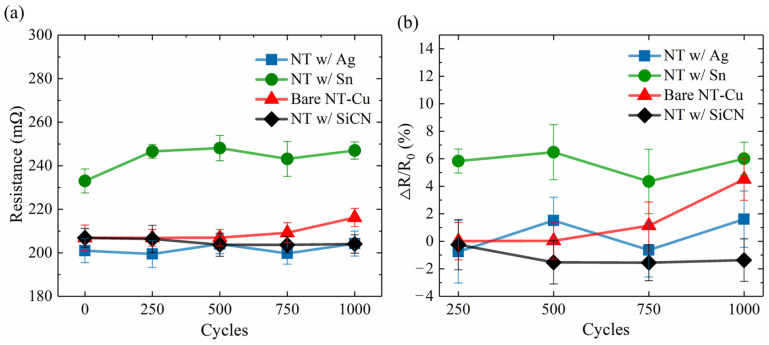
(**a**) Resistance of the RDLs before and after the TCT and (**b**) resistance change percentage of the RDLs after the TCT.

**Figure 5:**
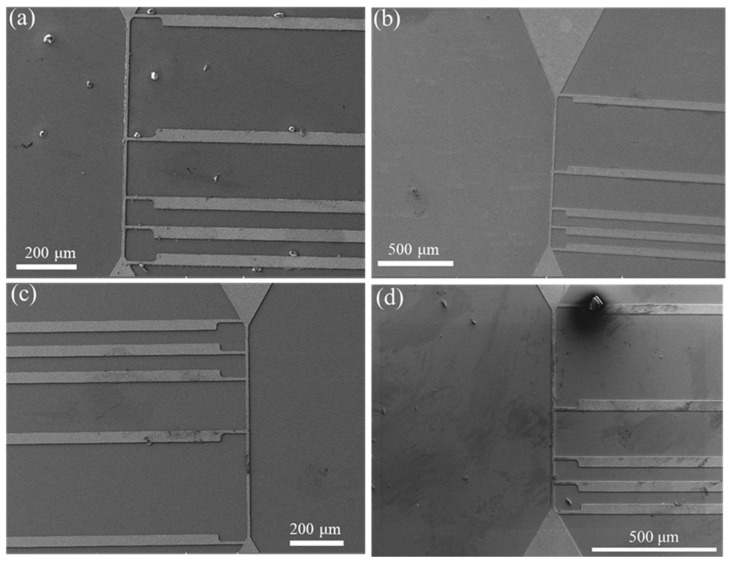
Plan-view SEM images of (**a**) the bare RDL sample and samples capped with (**b**) Ag, (**c**) Sn, and (**d**) SiCN after 1000 cycles of TCT.

The cross-sectional FIB micrographs of the as-passivated RDL samples are displayed in Figure 3. Three different kinds of passivation layers, each with a thickness of 100–200 nm, covered the top surface and both sidewalls of the NT-Cu line. Columnar nanotwinned structures were all observed in the Cu RDLs. The resistances of the RDLs before the TCT and after each 250 cycles of TCT are shown in Figure 4a. The initial resistance values of bare, Ag-passivated, Sn-covered, and SiCN-capped NT-Cu lines were 207, 201, 233, and 207 mΩ, respectively. The resistance changes, compared with the initial value and expressed in percentage, are presented in Figure 4b. For comparison, bare NT-Cu lines (without capping layer) were prepared as a control group. It can be seen that the resistance of the bare NT-Cu lines started to increase after 500 cycles and noticeably increased from 750 to 1000 cycles. The resistance rose by ~4.5% after 1000 cycles. On the contrary, both the Ag- and SiCN-capped NT-Cu lines showed only slight variations in resistance even after 1000 cycles. The Sn-coated NT-Cu lines exhibited the highest original resistance, and the resistance obviously increased within the first 250 cycles and remained stable thereafter. As shown in Figure 5, after 1000 cycles, no cracks or peeling were observed on the bare NT-Cu and passivated NT-Cu lines. This is because the high mechanical toughness of NT-Cu allows the RDLs to resist stress induced by temperature fluctuations [13]. The integrity of the NT-Cu lines eliminates the possibility that the resistance changes are due to line fractures.

**Figure 6:**
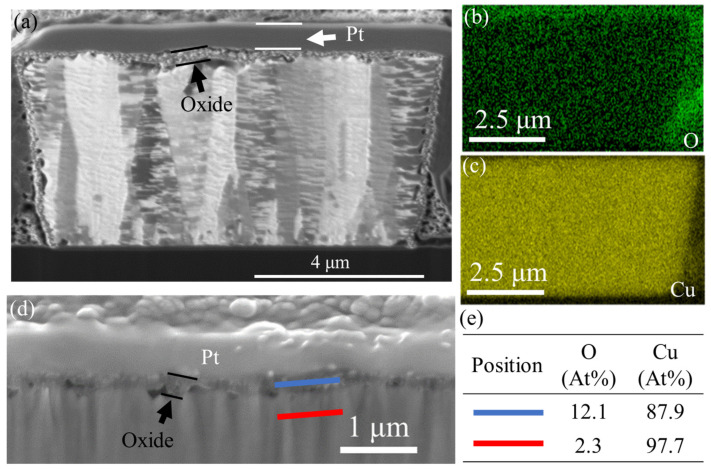
Failure analysis for the bare NT-Cu lines after 1000 cycles of TCT. (**a**) Cross-section of the bare NT-Cu line with EDX mapping of (**b**) oxygen and (**c**) copper elements; (**d**) top surface area of the bare NT-Cu line with EDX line-scanning positions; and (**e**) the corresponding atomic percent of the elements in the two positions.

Figure 6a shows the cross-sectional image of the bare NT-Cu RDL after 1000 cycles of TCT. The EDX analysis confirmed oxides on the periphery of bare NT-Cu RDL. As shown in the EDX mapping (Figure 6b,c), the oxygen signal was concentrated at the top surface and sidewall of the NT-Cu line. The EDX line scanning (Figure 6d,e) indicated that the oxygen signal of the oxide layer (blue line) was higher than that of the Cu line matrix (red line). Some voids were also found at the Cu/oxide interface. The self-diffusion coefficient of Cu ions is considerably higher than that of oxygen ions in Cu oxides. This difference primarily arises from the fact that Cu ions possess a much smaller radius compared to the oxygen ions found in Cu oxides. Therefore, the diffusion rate of outward Cu is significantly faster than that of inward oxygen in the oxide, which leads to vacancy aggregation around the Cu/oxide interface [34,35]. The formation of oxides and voids caused a resistance increase in the bare NT-Cu RDL after the TCT.

**Figure 7:**
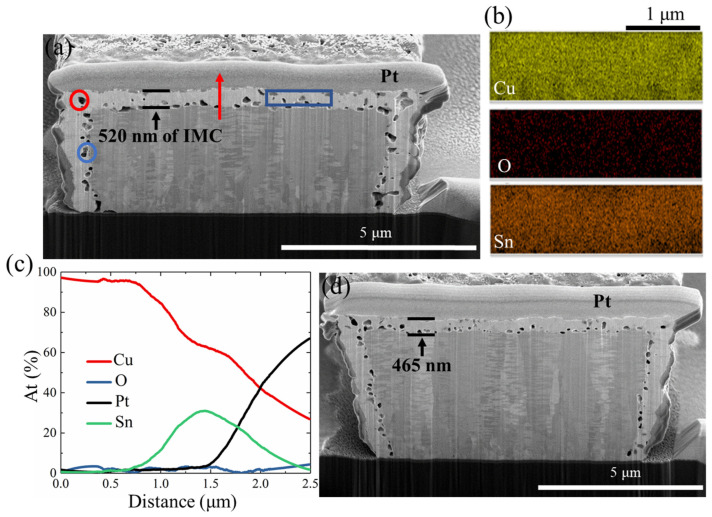
Failure analysis for the Sn-capped NT-Cu lines after TCT. (**a**) Cross-section of the Sn-capped NT-Cu line after 1000 cycles of TCT with (**b**) EDX mapping results of the blue rectangle area and (**c**) EDX line scan profile of the red arrow. (**d**) Cross-section of the Sn-capped NT-Cu line after 250 cycles of TCT.

Cu-Sn intermetallics (IMCs) and voids were found in the cross-sectional image of the Sn-covered NT-Cu RDL after 1000 cycles of TCT (Figure 7a). Figure 7b presents the EDX mapping results analyzed from the blue rectangle area in Figure 7a. The atomic percentages of Cu, Sn, and O were 68.7%, 28.4%, and 2.9%, respectively. The ratio of Cu to Sn was calculated to be 2.4, close to the elemental composition of Cu_3_Sn. This corresponds to Cu_3_Sn being considered the final phase of Cu-Sn IMC when the supply of Sn is limited [36,37]. During the Cu/Sn reaction, Cu_6_Sn_5_ initially forms, and then Cu_3_Sn is grown between the Cu_6_Sn_5_ layer and the Cu line. Before Cu_3_Sn grows to a certain thickness, the outward diffusion rate of Cu is greater than the inward diffusion rate of Sn, which causes Kirkendall void formation near the Cu/IMC interface [38], as marked by the blue circle in Figure 7a. The activation energies for the generation of Cu_3_Sn and Cu_6_Sn_5_ with the solid state are 38.7 and 47.3 kJ/mol, respectively [39]. Hence, voids away from the Cu/IMC interface (labeled by the red circle in Figure 7a) may result from volume shrinkage during the Cu_6_Sn_5_-Cu_3_Sn phase transformation, as described by the following chemical equation [38].

Cu_6_Sn_5_ → 2Cu_3_Sn + 3Sn (1)

After immersion in the Sn plating solution, Cu-Sn IMCs started to form on the surfaces of the NT-Cu line. The resistivities of Cu, Sn, Cu_6_Sn_5_, and Cu_3_Sn are 1.7, 11.0, 17.5, 8.3 × 10^−8^ Ω·m [25,40,41], respectively, at room temperature. The higher resistivities of Sn and IMCs contributed to the highest original resistance among the three passivation layers. Additionally, Cu reacts with Sn to form Cu_6_Sn_5_ and Cu_3_Sn first, and then the IMCs transform to Cu_3_Sn when Sn is depleted. Thus, the slight resistance drop at 750 cycles (Figure 4) may be attributed to the transformation of Cu_6_Sn_5_ into Cu_3_Sn. Furthermore, observing Sn-coated NT-Cu lines after 250 (Figure 7d) and 1000 cycles (Figure 7a), we found that the IMC thickness rose from 465 nm (Figure 7d) to 520 nm (Figure 7a). The IMC generation rate was highest within the first 250 cycles of TCT, which could explain the obvious resistance rise during this period and the relatively constant resistance afterward.

The aforementioned EDX mapping (Figure 7b) of the blue rectangle area in Figure 7a showed that the atomic proportion of oxygen was only 2.9%. Additionally, in Figure 7c, the line scan profile of the surface region, indicated by the red arrow in Figure 7a, shows that the Sn signal peaked when scanning over the Sn layer and then decreased, which resulted from the Sn concentration distribution along the scan path. The EDX line scanning (Figure 7c) also exhibited a low oxygen level throughout the surface region. These findings suggested that the Sn or IMC layers could effectively suppress the oxidation. Moreover, it is noted that the resistivity of Cu oxide (2.7 × 10^−3^ Ω·m) is about five orders larger than Cu (1.7 × 10^−8^ Ω·m) [25], whereas the resistivity of Cu_3_Sn (8.3 × 10^−8^ Ω·m) is approximately five times greater than Cu [41]. This signifies that Cu oxide has a more pronounced effect on the resistance changes compared to Cu_3_Sn. Although the generation of voids and IMCs led to comparatively higher resistance, the Sn layer helped to restrain the oxidation of Cu RDLs, thus enhancing their reliability. We can mitigate the increase in resistance by decreasing the thickness of the Sn coating layer, and therefore, the thickness of the Cu_3_Sn layer can also be reduced.

**Figure 8:**
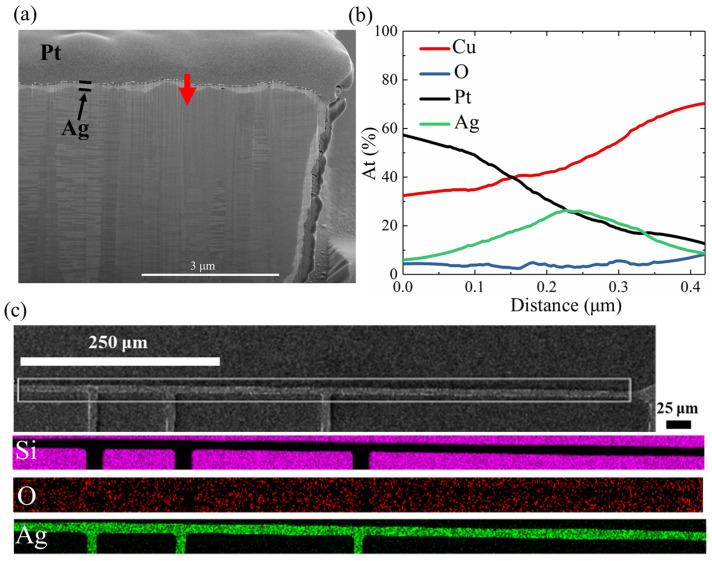
Failure analysis for the Ag-passivated NT-Cu lines after 1000 cycles of TCT. (**a**) Cross-section of the Ag-passivated NT-Cu line with (**b**) EDX line scan profile of the red arrow. (**c**) EDX mapping analysis of the plan-view Ag-passivated NT-Cu line.

**Figure 9:**
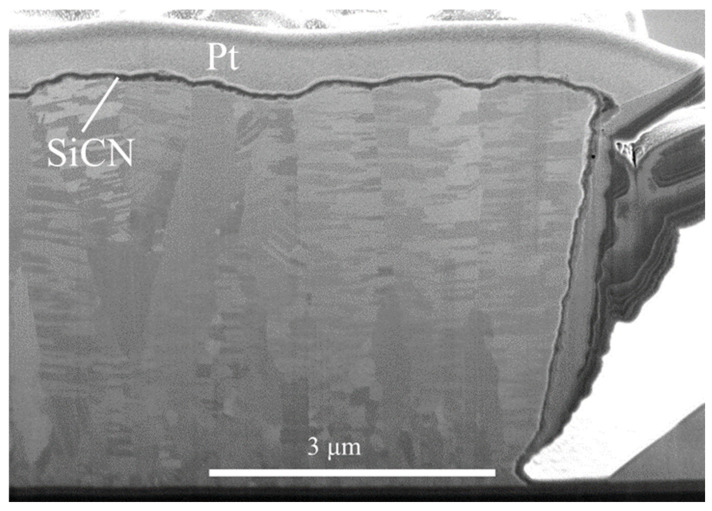
Cross-section of the SiCN-passivated NT-Cu line after 1000 cycles of TCT.

Figure 8a and Figure 9 present the FIB cross-sections of the Ag- and SiCN-passivated NT-Cu lines after 1000 cycles of TCT. No obvious changes in the microstructures were observed in the NT-Cu line coated with Ag (Figure 8a) or SiCN (Figure 9). Additionally, the EDX line scan profile near the Ag/Cu interface (labeled by the red arrow in Figure 8a) is depicted in Figure 8b, showing a silver signal peak and a low oxygen signal. In Figure 8b, as the scan moved across the Ag layer, the signal intensity initially rose, reaching a maximum when directly over the Ag layer, and then diminished as the scan continued beyond the layer. As presented in Figure 8c, the EDX mapping of the plan-view Ag-capped NT-Cu line after 1000 cycles also displayed that the oxygen signal was not concentrated on the RDL surface. These results suggested that no oxide layers were generated around the Cu surface. Therefore, the resistance of NT-Cu RDLs did not significantly change after the TCT under the protection of Ag or SiCN.

## 4. Conclusions

In summary, we successfully fabricated NT-Cu RDLs covered by immersion Ag/Sn or PECVD SiCN with a thickness of 100–200 nm under low operating temperatures (60 °C for immersion and 150 °C for PECVD). These surface-coating layers can adhere uniformly to the surfaces of NT-Cu RDL and provide effective blocking capability. The resistance variations, void formation, and oxidation of NT-Cu RDLs with different protective layers after the TCT were then investigated. The results indicated that the NT-Cu lines were not damaged after the TCT owing to their exceptional mechanical toughness. However, we observed a noticeable increase in resistance of the bare NT-Cu lines around 750–1000 cycles, with a 4.5% rise after 1000 cycles. This resistance change was attributed to the formation of oxides and voids. In contrast, both Ag and SiCN coatings effectively isolated the NT-Cu lines from oxygen and moisture and restrained the formation of oxides, leading to negligible changes in microstructure and resistance even after 1000 cycles of TCT. As for the Sn-coated NT-Cu RDLs, we found an apparent resistance rise within the first 250 cycles due to the fast formation of IMC. However, the resistance remained relatively stable after 250 cycles, and low oxygen signals were detected, which suggested that the Sn passivation layer effectively suppressed the oxidation of RDLs during the TCT. These results are significant for current technologies as they demonstrate the potential of low-temperature coatings to enhance the reliability of NT-Cu RDLs in electronic applications. This study provides facile fabrication processes of passivation layers with a low thermal budget, offering additional options to improve device performance and longevity. Future research directions may include exploring new materials for passivation layers and optimizing their performance under various conditions.

## Data Availability

The original contributions presented in the study are included in the article, further inquiries can be directed to the corresponding author.
